# A failed attempt to explain relative motion illusions via motion blur, and a new sparse version

**DOI:** 10.1177/20416695221124153

**Published:** 2022-09-15

**Authors:** Michael Bach

**Affiliations:** Eye Center, Medical Center, University of Freiburg, Germany Faculty of Medicine, University of Freiburg, Germany

**Keywords:** illusion, relative motion, contrast, blur, eye movement, Ouchi

## Abstract

Visual patterns can evoke marked, even beautiful motion illusions even if they are static; eye movements in all likelihood serve as temporal modulators. This paper concentrates on Ouchi-type “relative” or “sliding” motion illusions. It outlines an eye-motion-evoked motion-blur hypothesis, which does not correctly predict the shift direction of maximal illusion. This failure led to a nearly new particularly simple stimulus: an arrangement of dashed lines that strongly evokes a relative motion illusion, the “orthogonal dotted lines sway.” The latter is well explained by motion integration.

Certain static visual patterns can evoke strong motion illusions upon inspection. Different parts of the stimulus seem to move relative to each other, usually in a “jittery” fashion. An appropriate designation seems “relative motion illusion” (Hine et al., 1995); the most well-known example is the “Ōuchi illusion” (Hine et al., 1995, 1997; Pinna & Spillmann, 2005; Spillmann, 2013; Spillmann et al., 1986)^1^. There are many interesting variants (see below), and there seems to be no fully accepted explanation in the literature, although the motion integration hypothesis comes closest (Hine et al., 1997), elaborated and supported with data by Mather (2000). So the present paper concentrates on this type of relative motion illusion, trying—unsuccessfully—to explain it and presenting a new, very sparse, version.

Figure 1 depicts four different patterns that evoke the relative motion illusion. They share the following properties:

- The illusion basically consists of apparent segregation of the viewed pattern into two (or more) groups. These groups seem to move relative to each other, and—rule of common fate—thus segregate into disparate gestalten. The term “relative motion illusion” seems appropriate.
- With steady fixation no illusion occurs.
- Motion, especially irregular motion, of the presenting paper or screen, or head motion, evokes and/or amplifies the illusion.
- There are simple manipulations which modulate the illusion. An example is the “Spine Drift” illusion (Kitaoka, 2010; online: <http://www.psy.ritsumei.ac.jp/∼akitaoka/ECVP2010poster.jpg>), (Figure 2); there rotating one group of spines by 90° obliterates the illusion (see Bach (2011) for a demonstration of this).
- The illusion strength increases with luminance; for strongest effect it is suggested to view the examples at relatively high luminance; computer screens (laptop or desktop) typically now have 100 cd/m^2^ and more, which is enough.

**Figure 1.**
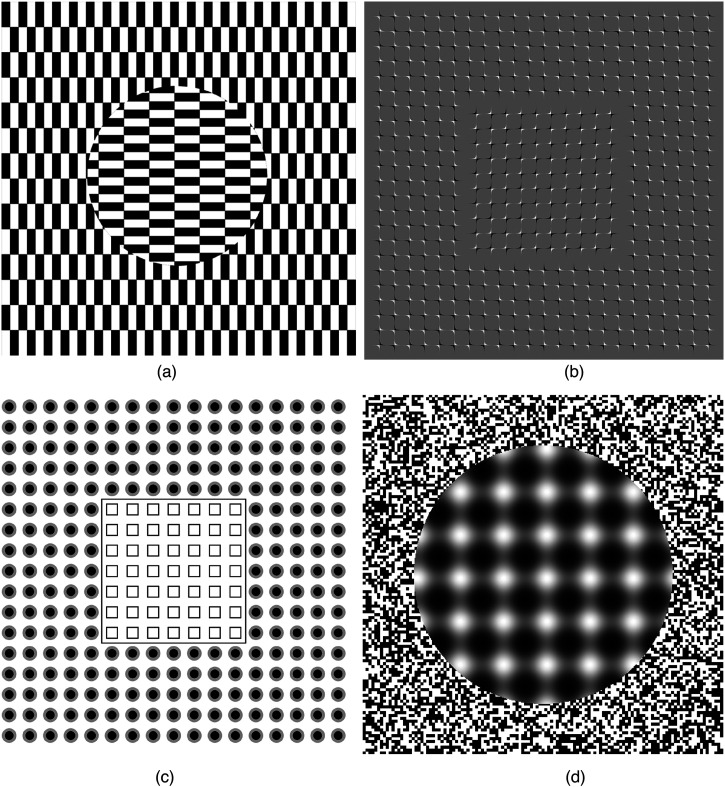
(a) Ōuchi illusion (Pinna & Spillmann, 2005). (Image from (Bach, 2022)). (b) “Spine Drift” illusion (Kitaoka, 2010, modified). (c) After Figure 1b from (Pinna & Spillmann, 2005). (d) “Out of Focus” (Kitaoka).

**Figure 2.**
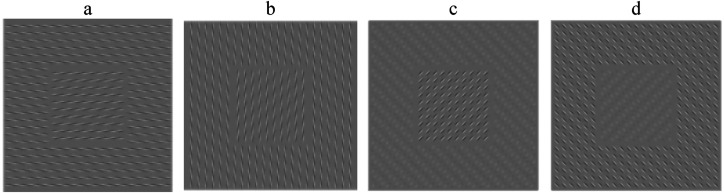
Effect of motion blur on the “drifting spines illusion” (Figure 1b). Here are motion blurred versions, blur size: 9 pixels. Motion direction: 0° (horizontal; a), 90° (vertical; b),  ± 45° (oblique; c, d). Because of the shape of the individual spines, motion blur at 0° and 90° blurs the spine in the inner square and the surround similarly; oblique motion blur leaves high texture contrast either at the surround or in the center.

In his review of the Ōuchi illusion, Spillmann (2013, p. 413) summarizes “… there is one phenomenon … but nine different attempts to account for it.” Motion blur is not among these candidates, although it could be seen as a specific case of “(i) involuntary eye movements interacting differentially with the two sets of checks, …” (Spillmann, 2013, p. 413). I initially hypothesized that this low-level explanation, motion blur, might account for relative motion illusions. As demonstrated below, it might account for the above commonalities, and led me to a new, very simple pattern, strongly evoking a relative motion illusion. It does not, however, explain the Ōuchi illusion after all. All the same, I will outline the hypothesis in basic steps which rely on known properties of the visual system, and then demonstrate that motion blur leads to differential local texture contrast, in turn causing local motion mis-assessment, which could be perceived as relative motion within the static picture.

## Motion-Blur Hypothesis to Explain the Relative Motion Illusion

I hypothesized that the following chain of mechanisms might be involved.

**Figure 3.**
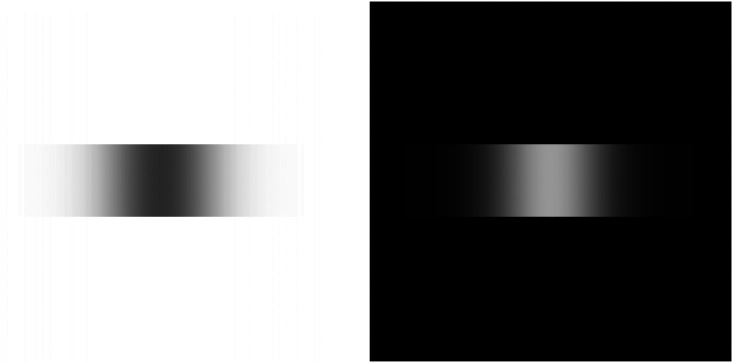
Effect of horizontal (0°) bidirectional motion blur on a square. On the left motion blur with a space parameter of 32 pixels was applied to a black 64 × 64 pixel square with white background; on the right the luminances were inverted. Note that horizontal blur leaves vertical structures unaffected.

1. Rapid movement of visual patterns on the retina leads to motion blur, which is perceptually suppressed (Burr & Morgan, 1997; Land, 1999).
2. Motion blur can affect different parts of the stimulus pattern differently. This is illustrated by applying directional, or anisotropic, blur (motion blur, Figure 3) to the stimulus pattern. Depending on the direction of the blur, there can occur modulation of local texture contrast, segregating the pattern based on common local contrast.
3. Contrast modulates perceived motion speed: reduction of contrast reduces perceived speed (Thompson, 1982).
4. Assumption: motion-evoked contrast modulation occurs at a neural stage before the blur effects are suppressed for conscious perception.
5. The above together lead to relative displacement of the neural correlate of the stimulus parts with different motion blur response causing the “jittery” percept.

**Figure 4.**
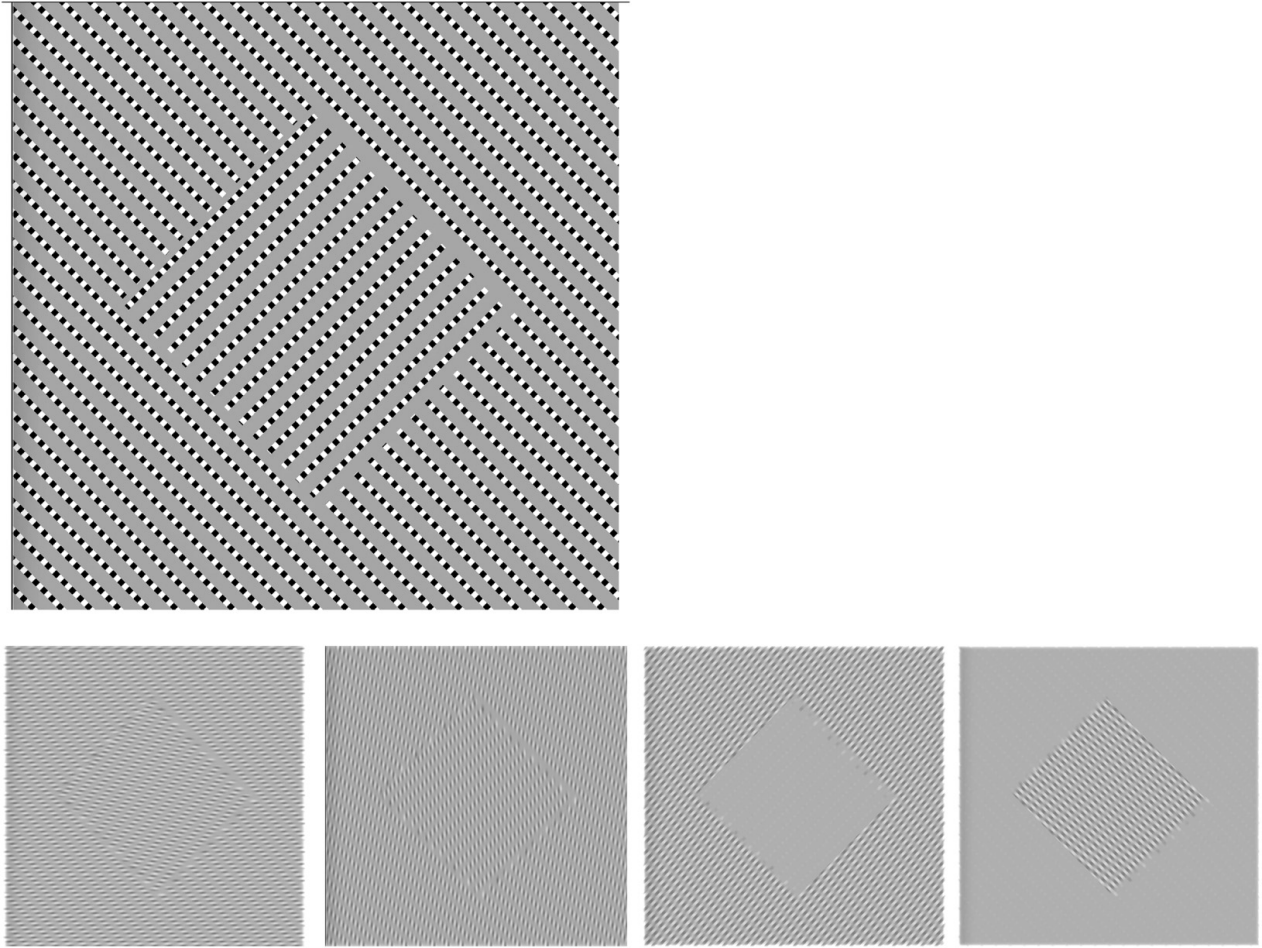
On top left is possibly the simplest relative motion illusion: the “orthogonal dotted lines sway.” Below are motion blurred versions, blur size 9 pixels. Left: motion blur 0° and 90° (horizontal and vertical); right: motion blur at  ± 45° (oblique). Because of the shape of the individual spines, motion blur at 0° and 90° blurs the spine in the inner square and the surround similarly; oblique motion blur leaves high texture contrast either at the surround or in the center (Bach, 2022, on-line with experimental controls).

In the following I will illustrate the above hypothesis by applying motion blur to the Ōuchi and Drifting Spines illusions. To further simplify the local shapes the model above predicted that a very simple pattern (“orthogonal dashed lines sway”) should display the motion illusion which indeed is the case (Figure 4).

## Methods

Bitmap-images were calculated using Cappuccino (Cappuccino – Modern App Development for the Web, 2010) and subjected to motion blur, as implemented in the application GraphicConverter (Lemke, 2021) version 11.

## Results

**Figure 5.**
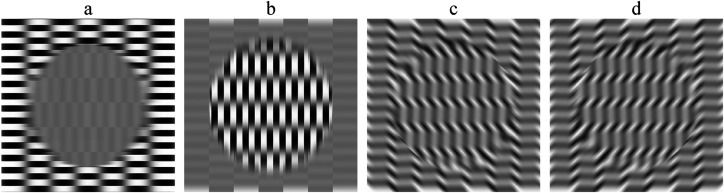
Motion blurred versions of the Ōuchi illusion (size: 1244 × 1244 pixels, blur size: 9 pixels). (a, b): Motion blur 0° and 90° (horizontal and vertical); (c, d): motion blur at  ± 45° (oblique). The blur will affect the rectangles to a smaller degree if acting along the long axis, and to a larger degree along the short axis. Thus motion blur at 0° and 90° affects the inner circle versus surround quite differently (a, b); oblique motion blur (c, d) affects the circle and the surround similarly. Depending on the direction of the motion blur, the mean texture contrast in the center or surround varies drastically.

Figure 5 exemplifies the effect of motion blur on the Ōuchi illusion (Figure 1a). Motion blur in four directions was applied. At 0° and 90° (horizontal and vertical; Figure 5a and b), motion blur affects the central circle and surround quite differently, leading to strong differences in texture contrast (the amount of motion blur was chosen to maximize the difference in local texture contrast). At  ± 45° (oblique; Figure 5c and d) orientations, the central circle and surround are fairly similarly affected, leading to markedly less difference between surround and central circle with respect to local texture contrast. However, this modulation in texture contrast cannot explain the Ōuchi illusion, because the illusion is strongest when the pattern is shifted obliquely at 45° (Mather, 2000) (seen here: Bach, 2022 {select Ōuchi Pattern}). Thus, the motion-blur hypothesis fails to predict the pattern-motion direction that evokes maximal illusion.

If we apply motion blur to the “Drifting Spines” illusion, we again find differential effects of the blur direction on the center and surround (Figure 2). As it happens, the choice for the orientation of the original “Drifting Spines” (Kitaoka, 2010) was such that cardinal blur directions (0° and 90°, horizontal and vertical; Figure 2a and b) lead to a fairly homogenous texture: All “spines” are reduced in contrast likewise, and the texture contrast is globally reduced. With motion blur set to  ± 45° (oblique; Figure 2c and d), given the specific shape and orientation of the spines, now the center and the peripheral ones are affected differentially. Consequently, either the surround (3rd image) or the central square (rightmost image) stand out in local texture contrast. Thus, the motion-blur model predicts oblique pattern shift as maximal illusion inducer. However, horizontal or vertical pattern shift clearly evokes a stronger illusion (Bach, 2011)—another failure of the motion-blur hypothesis.

There are a number of variations on the shape of the local elements “floating around the internet,” most taken from Kitaoka's pages <http://www.psy.ritsumei.ac.jp/~akitaoka/togetogedriftillusion.html>; many are quite intricate in shape. Thus, I pursued the question of which might be the *simplest* local shapes that would exhibit a relative motion illusion, working from the above hypothesis (at that time not yet realizing it was wrong) to maximize texture contrast for pattern shift. It turned out that simple dashed lines—each “dot” being square and alternatively black and white on a gray background (Figure 4, top, “orthogonal dotted lines sway”) will lead to an illusory strength quite similar to the “Drifting Spines” and its variations. The anisotropic action of motion blur acts on dashed lines quite differently along the line (strong contrast-reducing blur) as opposed to orthogonally (no contrast-reducing blur) (Figure 4). Again, the motion-blur hypothesis, while strongly modulating texture contrast, does not correctly predict the pattern shift direction for strongest illusion. But there are some interesting observations: (1) The background luminance is important: illusion is strongest when background luminance is the same as the spatial average across the pattern. (2) Equiluminance markedly reduces illusion strength. This (and more) can be manipulated in the on-line version (Bach, 2022).

## Discussion

While there is no question that motion blur affects the retinal image, this is an example that plausibility does not a proof make: Predictions from the motion blur hypothesis as outlined here do not fit experimental evidence when it comes to the pattern shift direction evoking maximal illusion. The irony is that its hypothesized mechanism (while I had not realized the failure) led to construction of the possibly most basic relative motion illusion (Figure 4, top). As a reviewer rightly pointed out, there is already a similar, if weaker version on Kitaoka's pages <http://www.psy.ritsumei.ac.jp/∼akitaoka/motion29e.html> (“Kite 2”). So I added a line width controller to (Bach, 2022) and it turns out that the illusion is stronger with thicker lines as used here.

The various relative motion illusions are phenomenologically quite similar—they all lead to the “swimming,” “sliding,” or “swaying” percept. They have in common that eye movements are involved (or other means that shift the pattern across the retina), which might lead to phenomenological similarity. Quite possibly, different mechanisms lead to the seeming differential motion percept (e.g., Ashida, 2002). For instance: equiluminance nearly extinguishes the orthogonal dotted-line sway (Figure 4) while the Ōuchi illusion is very little affected. Pinna and Spillmann (2005) suggest that the motion integration bias hypothesis (Hine et al., 1997), elaborated and supported with data by Mather (2000), accounts for the Ōuchi illusion; they also present “new illusions of sliding motion in depth,” “in which the elements have no oriented edges at all and yet elicit vivid apparent sliding motion” (see Figure 1c and d). These have in common a widely different spatial frequency range for the segregating patterns; so motion blur, acting as a spatial low pass, might play a role in explaining them.

The motion integration bias would also account for the orthogonal dotted-line sway (Figure 4, top). While the dotted line is made up of squares rather than rectangles in the Ōuchi illusion, the background luminance leads to asymmetry: A black-white edge evokes a stronger velocity vector than a black (or white)-grey edge; thus their vector sum leads to a motion-mis-estimate; this in turn causes a relative motion difference between the segregating patterns. Indeed, when one changes the background to white, the illusion disappears. Changing the white gaps in the dotted lines to grey causes reappearance of the illusion, though weak, but with opposite direction sign. This is predicted by the motion integration hypothesis; it does not even need the bias invoked by Mather (2000) for a more general explanation. Thus it is possible that the orthogonal dotted-line sway demonstrated here (Figure 4) is a variant of the “Illusory movement of dotted lines” (Ito et al., 2009)—there the background also plays a major role (Bach, 2015; interactive demo).

In summary, motion-blur might play a role for perceptual effects since it leads to marked modulation of texture contrast as shown here, and it might explain relative motion illusions without differentially oriented edges (Figure 1c and d).; it does not, however, correctly predict the shift direction causing maximal illusory motion for the Ōuchi illusion, nor the “Spine Drift” illusion, nor the dashed lines illusion developed here (Figure 4). Thus, while phenomenologically the various relative motion illusions appear very similar (shifting/swimming), possibly because they are all coupled to eye movements, the exact mechanisms are probably not the same for all relative motion illusions.

## Notes

1. A number of motion illusions from static images will not be considered here: (1) The “Rotating Snake illusion” (Atala-Gerard & Bach, 2017; Kitaoka, 2003; Kitaoka & Ashida, 2003), of which there are many variants, also involves eye movements, but its mechanism differs from the relative motion illusion: The Snakes-type illusion requires patterns with asymmetric luminance steps and seems satisfactorily explained as a natural consequence of arrays of basic motion detectors, coupled with a little saturating non-linearity (Bach & Atala-Gérard, 2020). (2) The “Pinna-Brelstaff” illusion (Pinna & Brelstaff, 2000) requires physical motion and evokes a motion percept perpendicular to the physical motion (see Bach (2003) for a demonstration). (3) “Enigma” (Léviant, 1996) with its periodic stripe arrangements is most likely due to interference with afterimages (Zanker & Walker, 2004) (see Bach (2014) for a demonstration). The percept is hazy poorly-localized motion; quite different from the figure-segregating phenomena addressed here.
